# Is parental monitoring just a way to acquire knowledge? Re‐evaluating a Re‐conceptualization

**DOI:** 10.1002/jcv2.70008

**Published:** 2025-03-18

**Authors:** Isabel R. Aks, John C. Schwarz‐Torres, Isabella S. Davis, Sarah J. Racz, Herry Patel, Oscar Gonzalez, William E. Pelham

**Affiliations:** ^1^ Department of Psychiatry University of California San Diego La Jolla CA USA; ^2^ Department of Psychology University of Maryland College Park MD USA; ^3^ Department of Psychology and Neuroscience University of North Carolina at Chapel Hill Chapel Hill NC USA

**Keywords:** adolescents, parental knowledge, parental monitoring

## Abstract

From the 1950s–1990s, parental monitoring was conceptualized and studied as a “socializing mechanism,” driving changes in youth adjustment via several channels. In the past 2 decades, parental monitoring has become re‐conceptualized in many papers as simply one of several ways to obtain parental knowledge, with knowledge replacing monitoring as the construct of central interest. This paper reviews literature showing that this re‐conceptualization is not supported by the extant empirical evidence and is strongly contradicted by theory, because parental monitoring may impact youth adjustment in several plausible ways that do not involve acquiring knowledge. As a result, we recommend the field change how it conceptualizes parental monitoring vis‐a‐vis parental knowledge. Just as we realized parental knowledge doesn't come *only* from parental monitoring, now we should realize that parental monitoring is not *only* a way to get parental knowledge. More evidence is needed to determine exactly how much of the effect of monitoring flows through increased knowledge and what other channels are in play.

**Key points**

**What's known?**

- Much research on parental monitoring today is guided by an implicit single‐mechanism assumption: that the only way monitoring affects youth adjustment is by first increasing parental knowledge.

**What's new?**

- We scrutinize this assumption and find it lacks theoretical or empirical support. There are several plausible mechanisms by which monitoring could improve adjustment without first resulting in increased knowledge.

**What's relevant?**

- As a result, research has not explored the full breadth of mechanisms underlying the protective effects of monitoring and may be missing opportunities to enhance or tailor clinical applications of the construct.

## A PARADIGM SHIFT: FROM PARENTAL MONITORING TO PARENTAL KNOWLEDGE

This paper diagnoses a shift that has occurred in the study of parental monitoring, identifies an implicit assumption underlying that shift, and shows that the assumption is neither theoretically nor empirically supported. As a result, the field may have prematurely de‐emphasized the study of important aspects of parental monitoring.

**FIGURE 1:**
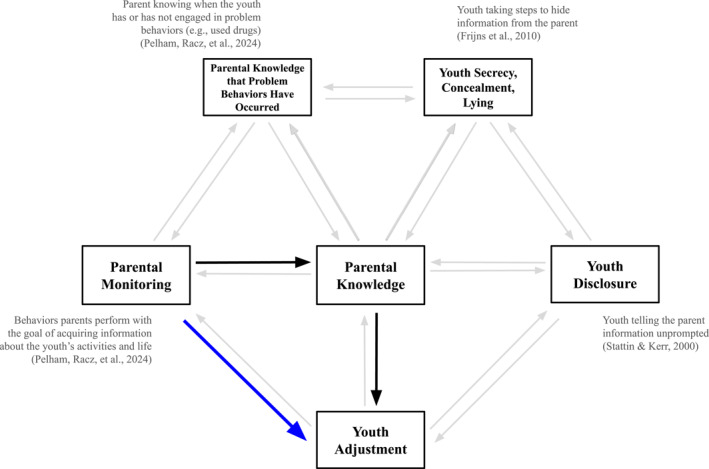
Parental Monitoring as One Element in the Parent‐Youth Monitoring Process. The figure is adapted from Pelham, Racz, et al. (2024) and depicts the parent‐youth monitoring process as a network of bidirectional, dyadic interactions among its elements. The list of included elements is not meant to be exhaustive, but to show how parental monitoring fits into this bigger picture. This argument in this paper focuses specifically on the three bolded paths shown between parental monitoring, parental knowledge, and youth adjustment.

*Parental monitoring* refers to the behaviors a parent performs with the goal of acquiring information about a youth's activities, whereabouts, companions, and life (Pelham, Racz, et al., 2024). Parental monitoring is just one element of a broader *parent‐youth monitoring process*: the continuous, dyadic interplay between caregivers and youth as they navigate caregivers' attempts to monitor youth (Pelham, Racz, et al., 2024). Many studies have shown that factors like youths' disclosure of information to parents, secretive behavior, and sense of privacy play important roles in the parent‐youth monitoring process (Frijns et al., 2010, 2020). Within the broader picture shown in Figure 1, the argument advanced in this paper focuses narrowly on one important link: how parental monitoring behaviors affect youth adjustment (i.e., the path colored in blue).

Historically, parental monitoring was regarded as an important construct due to its apparent associations with many facets of youth adjustment. In the past 20 years, a new wave of research emerged that instead put *parental knowledge* ‐ what parents actually know about their youth's life ‐ at the center of study (Frijns et al., 2020; Kerr & Stattin, 2000). The field shifted from examining parental monitoring as a determinant of youth adjustment to examining monitoring as a way to acquire parental knowledge.

Why did this shift occur? The genesis was a pair of seminal papers written by Stattin and Kerr (Kerr & Stattin, 2000; Stattin & Kerr, 2000), with results subsequently replicated and extended with longitudinal data (Kerr et al., 2010). Stattin and Kerr showed that nearly all studies ostensibly linking monitoring to youth adjustment had measured “monitoring” by asking questions about parental knowledge (Stattin et al., 2010). Because the empirical literature had actually been linking knowledge, not monitoring, to outcomes, Stattin and Kerr asked a question that naturally followed: where does parental knowledge come from? In two samples of adolescents in Sweden (Kerr & Stattin, 2000; Stattin & Kerr, 2000), they showed that parental knowledge was predicted more strongly by adolescents' voluntary disclosure of information than by parents' active monitoring to gather information. On this basis, they argued for re‐interpreting the parent‐youth monitoring process as driven in substantial part by youth, not parents, and that enhancing youth disclosure, rather than monitoring, might be what parents should focus on.

Stattin and Kerr's points were a much‐needed and welcome corrective for a field that had neglected the role youth play in the parent‐youth monitoring process. Their central claim—that most of what parents know about youths' lives comes from youth disclosing it—is one we agree with, and the relative size of disclosure versus monitoring effects is not a question addressed in this paper. Their findings spawned a burgeoning literature on “information management” (Frijns et al., 2020; Laird et al., 2018) that explored the process by which youth regulate parents' access to information. This literature has enumerated and studied links in the parent‐youth monitoring process that had been previously ignored (Figure 1) and left the field with a far richer understanding of how parents and youth navigate the seeking and sharing of information.

Yet the positive thrust of Stattin and Kerr's re‐interpretation had an inadvertent and subtle side effect. As the monitoring *process* was re‐interpreted, the parental monitoring *construct* was also implicitly re‐conceptualized. Before Stattin and Kerr (2000), parental monitoring had been conceptualized as a multidimensional family management practice that promoted healthy youth adjustment in several ways (Dishion & McMahon, 1998). Monitoring was a “socializing mechanism” (Patterson, 1982, p. 222): it meant not only setting expectations and tracking behavior but establishing regular times for family discussion, expressing interest in the youth's life and well‐being, and building emotional attachments among family members. In other words, monitoring was just not about the knowledge it brings but the messages it sends, the feelings it creates. Yet after Stattin and Kerr (2000), parental monitoring came to be conceptualized quite differently: simply as one of several ways to get knowledge, no different structurally than getting the same information disclosed voluntarily by the youth. Stattin and Kerr didn't argue for this shift directly; it simply fell out of their analysis, of turning the primary question of the field into, “Where does knowledge come from?”

This paper scrutinizes the implicit assumption underlying the subtle re‐conceptualization of parental monitoring we are describing. Namely, the assumption *that the only way parental monitoring* *can* *affect youth adjustment is by first increasing parental knowledge*. If that implicit assumption is true, then focusing the study of parental monitoring on how it yields knowledge is a sensible reaction to Stattin and Kerr's findings. But if that implicit assumption is false, then focusing the study of parental monitoring on how it yields knowledge could steer the field away from important questions about how monitoring works and affects youth adjustment *without* involving knowledge. The next section evaluates this implicit assumption with empirical evidence and theory.

## IS THE ONLY PURPOSE OF PARENTAL MONITORING TO GET KNOWLEDGE?

### What do empirical studies find?

Do empirical studies support the implicit assumption that monitoring affects youth adjustment only by first increasing parental knowledge? In short, no. We can start by considering Stattin and Kerr's initial results, which provide evidence to the contrary. Kerr and Stattin (2000) reported a series of regressions of measures of youth outcomes on parental monitoring while adjusting for parental knowledge. In 9 different tests, the association between monitoring and youth adjustment remained statistically significant when holding the amount of parental knowledge fixed. Thus, Kerr and Stattin's cross‐sectional data suggest that monitoring has effects on youth outcomes that do not flow entirely through knowledge.

**FIGURE 2:**
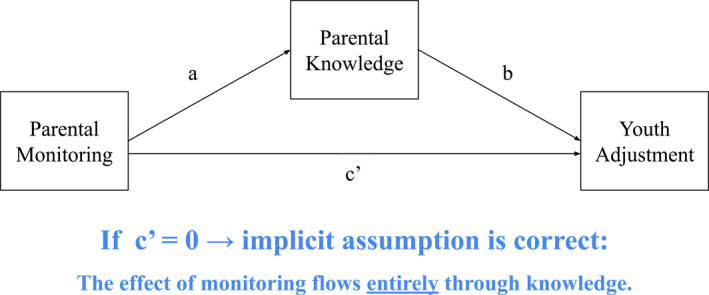
Testing the Implicit Assumption by Testing Statistical Mediation. Figure 2 depicts a mediation model of the relationship between parental monitoring and a youth adjustment measure, mediated by parental knowledge. The c’ path in this model represents the direct effect of parental monitoring on youth adjustment.

A natural way to test the assumption that parental monitoring affects youth adjustment only via increasing knowledge would be to perform a mediation analysis, as shown in Figure 2. Suppose that when parental knowledge is tested as a mediator, there is an indirect effect of monitoring (i.e., *ab* ≠ 0) but no direct effect (i.e., *c*’ = 0). That finding would suggest that monitoring affects youth adjustment only by increasing parental knowledge, as stated in the implicit assumption. Conversely, suppose that when parental knowledge is tested as a mediator, there is an indirect effect (i.e., *ab* ≠ 0) but also a direct effect of monitoring not mediated by knowledge (i.e., *c*’ ≠ 0). That finding would suggest that knowledge is only part of the reason that parental monitoring and youth adjustment are associated, falsifying the implicit assumption. If a direct effect remains, there must be other mediating mechanisms besides knowledge that are missing from the picture.

We searched for empirical studies that have fit the model shown in Figure 2, testing whether parental knowledge mediates the association between parental monitoring and youth adjustment. Specifically, we required that the study (1) include a sample of children (ages ≤ 18 years old), (2) measure parental monitoring as an action the parent takes (i.e., not conflate monitoring with knowledge, and (3) perform a statistical mediation analysis. We searched electronic databases, contacted the authors of every qualifying article, and asked prominent scholars in the field of parental monitoring if they knew of other studies that have tested this mediational chain (Figure 2).

Three studies published since Stattin and Kerr (2000) have tested whether knowledge mediates the link between monitoring and adjustment. These studies are summarized in Table 1 and together, the findings do not support a full mediation model. The indirect effect via knowledge was statistically significant in only about half of tests (7 out of 16). Furthermore, in about half of those tests with significant indirect effects (3 of 7), the direct effect of monitoring remained statistically significant. Turning to effect size, in no case did the estimated effect sizes suggest full mediation. The percent of the total effect of monitoring mediated by parental knowledge ranged from 6% to 34% ‐ suggesting that the remaining 66%–94% of the effect of monitoring flowed through some other mediating mechanism(s).

**TABLE 1:** Tests of statistical mediation by parental knowledge in published studies.

Sample description	Design	Findings	Details of tests				
**Criss et al.,** 2015	**Cross‐sectional**	**Direct** effect of monitoring (not via knowledge) was statistically significant in 1 of 6 tests (*p* < 0.05)		**a**	**b**	**c’**	**%** **m** **ediated**
*N* = 206 youth ages 10–18 years in low‐income families		**Indirect** effect of monitoring via knowledge was statistically significant in 0 of 6 tests	**Antisocial behavior**	Sol^a^: 0.01	Sol: −0.36*	Sol: 0.05	‐
				Inv^b^: 0.18	Inv: −0.36*	Inv: −0.10	6%
Recruited from disadvantaged communities through fliers.			**Substance use**	Sol: 0.00	Sol: −0.12	Sol: 0.06	‐
				Inv: 0.17	Inv: −0.12	Inv: −0.25*	8%
51% female			**School grades**	Sol: 0.01	Sol: 0.29***	Sol: −0.17	‐
				Inv: 0.17	Inv: 0.29***	Inv: 0.15	25%
27% white, 32% black, 19% Latino American							
**Fletcher et al.,** 2004	**Cross‐sectional analysis**	**Direct** effect of monitoring (not via knowledge) was statistically significant in 2 of 8 tests (*p* < 0.05)					
Sample 1 (S1):	Monitoring @ *T* = 1			**a**	**b**	**c’**	**% mediated**
*N* = 3998 adolescents	Knowledge @ *T* = 1		**Substance use**	Cross‐sectional	Cross‐sectional	Cross‐sectional	
Recruited from high schools	Outcomes @ *T* = 1			S1^c^: 0.31**	S1: −0.28**	S1: 0.14**	‐
				S2^d^: 0.38**	S2: −0.30**	S2: 0.17**	‐
Sample 2 (S2):	**Longitudinal analysis (T = 2)**	**Indirect** effect of monitoring via knowledge was statistically significant in 5 of 8 tests (*p* < 0.05)		Longitudinal	Longitudinal	Longitudinal	
Subset of sample 1	*Two timepoints spaced 1 year apart*.			S1: 0.31**	S1: −0.08**	S1: Not reported	‐
				S2: 0.38**	S2: −0.03	S2: Not reported	‐
*N* = 2568 adolescents	Monitoring @ *T* = 1		**Delinquency**	Cross‐sectional	Cross‐sectional	Cross‐sectional	
54% female, 66% non‐Hispanic white, 16% Asian, 11% Hispanic, 7% black	Knowledge @ *T* = 1			S1: 0.31**	S1: −0.16**	S1: Not reported	‐
	Outcomes @ *T* = 2			S2: 0.38**	S2: −0.27**	S2: Not reported	‐
				Longitudinal	Longitudinal	Longitudinal	
				S1: 0.31**	S1: Not reported	S1: Not reported	‐
				S2: 0.38**	S2: Not reported	S2: Not reported	‐
			***Coefficients*:** Standardized				
**Lippold et al.,** 2014	**Longitudinal (T = 3)**	**Direct** effect of monitoring (not via knowledge) was statistically significant in 1 of 2 tests (*p* < 0.05)		**a**	**b**	**c’**	**% mediated**
*N* = 780 youth in 6th grade	*Three timepoints;*		**Substance use**	0.29***	−0.12*	−0.20***	15%
Recruited from schools in rural communities	*T* = *1 and T* = *2 spaced 6 months apart, T* = *2 and T* = *3 spaced 2 years apart.*		**Delinquency**	0.29***	−0.07*	−0.04	34%
47% male	Monitoring @ *T* = 1	**Indirect** effect of monitoring via knowledge was statistically significant in 2 of 2 tests (*p* < 0.05)	***Coefficients:*** Unstandardized				
	Knowledge @ *T* = 2						
84% white, 6% Hispanic, 3% Black	Outcomes @ *T* = 3						

*Note*: In “Design” column, “T” = timepoint. a, b, c paths correspond to the model shown in Figure 2. In “Details of tests” column, percent mediated is calculated as $ab\;/\;\left(c^{\prime }+ab\right).$ Percent mediated is omitted for instances when ab and $c^{\prime }$ have opposite signs, when the metric is not readily interpretable (Preacher & Kelley, 2011). In all studies, parental monitoring assessed actions the parent took to acquire information about their child. Items in the knowledge scales for each article measured parental knowledge, or the extent to which the parent is aware of the youth's life.

***p < 0.001, **p < 0.01, *p < 0.05.

^a^ Parental solicitation is a predictor for all the results preceded by “Sol”.

^b^ Parental involvement is a predictor for results preceded by “Inv”.

^c^ Results for Sample 1 are preceded by “S1”.

^d^ Results for Sample 2 are preceded by “S2”.

First, Criss et al. (2015) tested parental knowledge as a mediator of the relation between monitoring and youth adjustment using a cross‐sectional sample of disadvantaged families in the U.S. (*N* = 206). The indirect effect was not statistically significant in any model across three different outcomes. As an effect size, the percent mediated ranged from 6% to 25%.

Second, Fletcher et al. (2004) explored mediation using two participant samples: a community‐based sample of 3998 high schoolers in the U.S., and 2568 adolescents who were a subset of this group. Fletcher et al. performed the analysis two ways: with the youth adjustment measure assessed concurrently to monitoring and knowledge, then with the youth adjustment measure assessed 1 year later. Their findings showed statistically significant direct effects of monitoring on substance use, but not delinquency.

Finally, Lippold et al. (2014) is the only published study to test whether parental knowledge mediates the association between monitoring and youth adjustment using three waves of longitudinal data, with monitoring, knowledge, and youth adjustment measured successively. Data were collected on a rural community‐based sample of 780 families. Parental monitoring was measured during the Fall of 6th grade, parental knowledge was measured during the Spring of 6th grade, and youth adjustment was measured during the Spring of 8th grade. As for Fletcher et al., the direct effect of monitoring was statistically significant for substance use, but not delinquency. The indirect effect of monitoring via knowledge was statistically significant for both substance use and delinquency, with the percent mediated being 15% and 34% respectively.

These tests have not been perfect. Criss et al. (2015) was entirely cross‐sectional, like Stattin and Kerr's studies (2000; 2000), and Fletcher et al. (2004) was partially cross‐sectional, measuring monitoring and knowledge at the same wave. Cross‐sectional data pose well‐known threats to the validity of mediation analyses because they cannot confirm the temporal precedence implied by mediation (Cole & Maxwell, 2003). To draw conclusions from mediation analyses, the predictor, mediator, and outcome variables should be temporally ordered. Only Lippold et al. (2014) fulfill this criterion, measuring monitoring, knowledge, and youth adjustment at three successive waves. Thankfully, Lippold et al. supports the same conclusions as the other two studies: that the effect of monitoring does not flow entirely through knowledge, with knowledge mediating only 15%–34% of the effect.

In summary, strikingly few studies—three—have tested the implicit assumption guiding much research on parental monitoring today and taken as a whole, their findings contradict it. Across all of the tests performed, only 6%–34% of the effect of monitoring on youth adjustment flowed through knowledge. These studies suggest knowledge is a mediator of monitoring's effects on youth adjustment, but not the only one—there must be missing mechanisms that can explain the remaining 66%–94% of the total effect.

### What does theory say?

**FIGURE 3:**
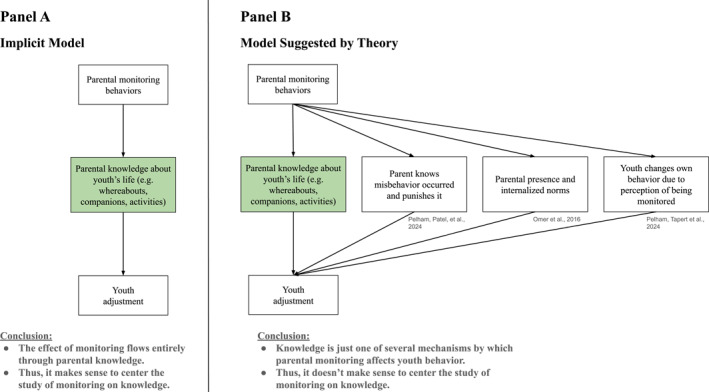
The Field's Implicit Single‐Mediator Model versus A Multi‐Mediator Model Suggested by Theory. Figure 3A depicts a model of parental monitoring where monitoring can only influence youth adjustment by first increasing parental knowledge ‐ this is posited to be an implicit assumption underlying why parental monitoring would be examined only in relation to parental knowledge. Panel B depicts a model of parental monitoring where monitoring operates through multiple routes to impact youth adjustment ‐ this model counters the implicit assumption and is empirically and theoretically suggested.

Like the empirical evidence, theory strongly contradicts the implicit assumption that monitoring effects flow entirely through increased knowledge. Published work articulates several mechanisms besides knowledge that could account for the residual direct effect of monitoring on adjustment found in the empirical literature reviewed above (Figure 3).

Pelham, Patel, et al. (2024) and Pelham, Tapert, et al. (2024) reviewed published theoretical models of how monitoring affects adjustment. Of the three reviewed theories (Hayes et al., 2003; Jaccard et al., 2010; Omer et al., 2016), not one proposed that parental monitoring's influence on youth adjustment could be explained fully, or even mostly, by the acquisition of parental knowledge. Hayes et al. and Jaccard et al. proposed that monitoring affects adjustment by helping parents find out when the youth has misbehaved and then delivering a punishment ‐ not by acquiring general knowledge about the youth's life. Omer et al. proposed that monitoring affects adjustment by inducing a “parental presence” in the youth's mind, increasing the salience of the parents' norms about behavior.

Parental monitoring could also influence the youth's perception of being monitored, which could change the youth's behavior without any accompanying increase in parental knowledge (Fletcher et al., 2004). For example, suppose a parent has turned on a smartphone feature that lets them track their youth's location, but they never actually open the app to check where their youth is. Though the parent is never gaining any knowledge about their youth, the youth simply being aware that the parent *could* check their location  may make the youth less likely to go to places where they would engage in delinquent behavior. Likewise, whether or not a parent's attempts to meet the parents of the youth's friends are successful, they send youth a message.

As another example, parental monitoring could give a parent incorrect knowledge—information that paints an incomplete picture or can be misinterpreted—which could provoke parental action that influences youth's behavior. For example, a parent can make rules under unfounded suspicions of their youth engaging in problem behavior, thus reducing the youth's future likelihood of engaging in problem behavior.

In summary, consistent with the empirical literature, theory does not support the assumption depicted in Figure 3A: that monitoring affects youth adjustment only by first increasing parent's general knowledge about the youth's life. Rather, existing theory suggests the existence of many additional channels by which parental monitoring could influence youth adjustment, as depicted in Figure 3B.

### What next?

Tracing the historical path of research on parental monitoring, this paper made three observations:

1. Spurred by Stattin and Kerr's seminal work (2000; 2000), the field shifted to re‐conceptualize parental monitoring as simply a way to obtain knowledge, rather than an independent determinant of youth adjustment.
2. The validity of that re‐conceptualization hinges on the implicit assumption that *parental monitoring affects youth adjustment only by first increasing parental knowledge.*
3. Theory strongly contradicts that implicit assumption and the extant empirical evidence does not support it. In the only 3 published tests we could locate, knowledge mediated only 6%–34% of the association between monitoring and adjustment.

We conclude that the field has re‐oriented its study of monitoring based on an assumption about the relation between parental monitoring and parental knowledge that might be wrong. More rigorous and comprehensive empirical work is needed to determine exactly how much of the effect of monitoring on adjustment flows through knowledge, and, if this percentage is as far from 100% as it appears today (Table 1), which other channels it flows through. The answer is important for several reasons.

The first is that judging monitoring based on how much it increases knowledge could be underestimating monitoring's importance. If knowledge is only one of several ways monitoring affects youth adjustment, then centering the study of monitoring around knowledge could be missing important elements of how monitoring works and why it matters.

The second reason is that figuring out what comprises “good” monitoring requires first understanding what target mechanisms monitoring is intended to change. Monitoring can be beneficial or harmful in different contexts (Laird et al., 2018), so both parents and clinicians are seeking ways to figure out when it's working and how it should be performed. If the goal of monitoring is to acquire knowledge, then beneficial monitoring will be that which results in knowledge; in contrast, if the goal of monitoring is to induce a sense of parental presence, or to inculcate a perception of being monitored (Figure 3), then beneficial monitoring might look entirely different.

The third reason is that today, monitoring runs the risk of being subsumed fully into research on parental knowledge. Scientific energy in the 25 years since Stattin and Kerr (2000) has been focused on knowledge, and the most cutting‐edge and novel work today is focused on how youth manage and share information, not the behaviors that parents perform. Is monitoring simply one of several ways to get knowledge, to be parsimoniously subsumed within the growing literature on youth information management? Or is monitoring more than that, deserving of attention as an distinct phenomenon and determinant of youth adjustment? Pending more definitive evidence, we recommend the field take the latter view. Just as we realized parental knowledge doesn't come only from parental monitoring, now we should realize that parental monitoring is not only a way to get parental knowledge. We expect a science that explores how monitoring and knowledge function independently and interactively to yield the most complete picture with the greatest impact.

## AUTHOR CONTRIBUTIONS

**Isabel R. Aks**: Conceptualization; formal analysis; methodology; project administration; supervision; writing—original draft; writing—review and editing. **John C. Schwarz‐Torres**: writing—original draft; writing—review and editing. **Isabella S. Davis**: writing—review and editing. **Sarah J. Racz:** writing—review and editing. **Herry Patel**: funding acquisition; writing—review and editing. **Oscar Gonzalez**: methodology; writing—review and editing. **William E. Pelham III**: conceptualization; funding acquisition; project administration; writing—review and editing.

## CONFLICT OF INTEREST STATEMENT

The authors declare no conflicts of interest.

## ETHICAL CONSIDERATIONS

This was a theoretical review article and did not collect new data.

## Data Availability

Data sharing not applicable to this article as no datasets were generated or analyzed during the current study.

## References

[jcv270008-bib-0001] Cole, D. A. , & Maxwell, S. E. (2003). Testing mediational models with longitudinal data: Questions and tips in the use of structural equation modeling. Journal of Abnormal Psychology, 112(4), 558–577. 10.1037/0021-843X.112.4.558 14674869

[jcv270008-bib-0002] Criss, M. M. , Lee, T. K. , Morris, A. S. , Cui, L. , Bosler, C. D. , Shreffler, K. M. , & Silk, J. S. (2015). Link between monitoring behavior and adolescent adjustment: An analysis of direct and indirect effects. Journal of Child and Family Studies, 24(3), 668–678. 10.1007/s10826-013-9877-0 25750505 PMC4349437

[jcv270008-bib-0003] Dishion, T. J. , & McMahon, R. J. (1998). Parental monitoring and the prevention of child and adolescent problem behavior: A conceptual and empirical formulation. Clinical Child and Family Psychology Review, 1(1), 61–75. 10.1023/A:1021800432380 11324078

[jcv270008-bib-0004] Fletcher, A. C. , Steinberg, L. , & Williams‐Wheeler, M. (2004). Parental influences on adolescent problem behavior: Revisiting Stattin and Kerr. Child Development, 75(3), 781–796. 10.1111/j.1467-8624.2004.00706.x 15144486

[jcv270008-bib-0005] Frijns, T. , Keijsers, L. , Branje, S. , & Meeus, W. (2010). What parents don’t know and how it may affect their children: Qualifying the disclosure–adjustment link. Journal of Adolescence, 33(2), 261–270. 10.1016/j.adolescence.2009.05.010 19573902

[jcv270008-bib-0006] Frijns, T. , Keijsers, L. , & Finkenauer, C. (2020). Keeping secrets from parents: On galloping horses, prancing ponies and pink unicorns. Current Opinion in Psychology, 31, 49–54. 10.1016/j.copsyc.2019.07.041 31454683

[jcv270008-bib-0007] Hayes, L. , Hudson, A. , & Matthews, J. (2003). Parental monitoring: A process model of parent‐adolescent interaction. Behaviour Change, 20(1), 13–24. 10.1375/bech.20.1.13.24844

[jcv270008-bib-0008] Jaccard, J. , Guilamo‐Ramos, V. , Bouris, A. , & Dittus, P. (2010). A three‐process system of parental monitoring and supervision. In J. Jaccard , V. Guilamo‐Ramos , & P. Dittus (Eds.), Parental monitoring of adolescents (pp. 176–204). Columbia University Press. https://www.jstor.org/stable/10.7312/guil14080.12

[jcv270008-bib-0009] Kerr, M. , & Stattin, H. (2000). What parents know, how they know it, and several forms of adolescent adjustment: Further support for a reinterpretation of monitoring. Developmental Psychology, 36(3), 366–380. 10.1037/0012-1649.36.3.366 10830980

[jcv270008-bib-0010] Kerr, M. , Stattin, H. , & Burk, W. J. (2010). A reinterpretation of parental monitoring in longitudinal perspective. Journal of Research on Adolescence, 20(1), 39–64. 10.1111/j.1532-7795.2009.00623.x

[jcv270008-bib-0011] Laird, R. D. , Zeringue, M. M. , & Lambert, E. S. (2018). Negative reactions to monitoring: Do they undermine the ability of monitoring to protect adolescents? Journal of Adolescence, 63(1), 75–84. 10.1016/j.adolescence.2017.12.007 29275081

[jcv270008-bib-0012] Lippold, M. A. , Greenberg, M. T. , Graham, J. W. , & Feinberg, M. E. (2014). Unpacking the effect of parental monitoring on early adolescent problem behavior. Journal of Family Issues, 35(13), 1800–1823. 10.1177/0192513X13484120 25382891 PMC4222511

[jcv270008-bib-0013] Omer, H. , Satran, S. , & Driter, O. (2016). Vigilant care: An integrative reformulation regarding parental monitoring. Psychological Review, 123(3), 291–304. 10.1037/rev0000024 26845385

[jcv270008-bib-0014] Patterson, G. R. (1982). Coercive family processes (Vol. 3). Castalia Publishing Company.

[jcv270008-bib-0015] Pelham III, W. E. , Racz, S. J. , Davis, I. S. , Aks, I. R. , Patel, H. , McMahon, R. J. , Thornburg, M. A. , Huang, Y.‐T. W. , Schulze, E. M. , Gonzalez, O. , Tapert, S. F. , & Brown, S. A. (2024). What is parental monitoring? Clinical Child and Family Psychology Review, 27(2), 576–601. 10.1007/s10567-024-00490-7 38869680 PMC11801412

[jcv270008-bib-0016] Pelham III, W. E. , Patel, H. , Somers, J. A. , & Racz, S. J. (2024). Theory for how parental monitoring changes adolescent behavior. Clinical Psychological Science, 13(1), 18–42. 10.1177/21677026241232926 39931200 PMC11810127

[jcv270008-bib-0017] Pelham III, W. E. , Tapert, S. F. , Gonzalez, M. R. , Ahiarakwe, U. , Patel, H. , Davis, I. S. , Meruelo, A. , Van Rinsveld, A. M. , Marshall, A. T. , Dick, A. S. , Guillaume, M. , Dowling, G. J. , Baskin‐Sommers, A. , & Brown, S. A. (2024). How does parental monitoring reduce adolescent substance use? Preliminary tests of two potential mechanisms. Journal of Studies on Alcohol and Drugs, 85, (3), jsad.23–00297. 10.15288/jsad.23-00297 PMC11095493 38227391

[jcv270008-bib-0018] Preacher, K. J. , & Kelley, K. (2011). Effect size measures for mediation models: Quantitative strategies for communicating indirect effects. Psychological Methods, 16(2), 93–115. http://dx.doi.org.ezproxy1.lib.asu.edu/10.1037/a0022658 21500915 10.1037/a0022658

[jcv270008-bib-0019] Stattin, H. , & Kerr, M. (2000). Parental monitoring: A reinterpretation. Child Development, 71(4), 1072–1085. 10.1111/1467-8624.00210 11016567

[jcv270008-bib-0020] Stattin, H. , Kerr, M. , & Tilton‐Weaver, L. C. (2010). Parental monitoring: A critical examination of the research. In V. Guilamo‐Ramos , J. Jacquard , & P. Dittus (Eds.), Parental monitoring of adolescents: Current perspectives for researchers and practitioners (pp. 3–38). Columbia University Press.

